# Construction and Evaluation of a Novel MAP Immunoassay for 9 Nutrition-and-Health-Related Protein Markers Based on Multiplex Liquid Protein Chip Technique

**DOI:** 10.3390/nu15061522

**Published:** 2023-03-21

**Authors:** Jiyong Yin, Jiangping Niu, Junsheng Huo, Jing Sun, Jian Huang, Chaoqun Sun

**Affiliations:** Key Laboratory of Trace Element Nutrition of National Health Commission of the People’s Republic of China, National Institute for Nutrition and Health, Chinese Center for Disease Control and Prevention, Beijing 100050, China; yinjy@ninh.chinacdc.cn (J.Y.);

**Keywords:** liquid protein chip, nutrition and health, protein marker, multiplex detection

## Abstract

We attempted to construct and evaluate a novel detection method to realize simultaneous detection based on a multiplex liquid protein chip technique for nine nutrition-and-health-related protein markers to meet the requirement of an accurate, simultaneous and comprehensive analysis of the proteomics of nutrition and health. The lower limits of detection, biological limits of detection and regression equations of serum ferritin (SF), soluble transferrin receptor (sTfR), c-reactive protein (CRP), retinol-binding protein4 (RBP4), apolipoprotein B (ApoB), alpha-fetoprotein (AFP), prealbumin (PA), carcino-embryonic antigen (CEA) and D-Dimmer (D-D) were determined after a series of optimal experiments. Then, the results of the methodological evaluation for this novel method indicated that the accuracies were between 70.12% and 127.07%, the within-run precisions were between 0.85% and 7.31%, the between-run precisions were between 3.53% and 19.07%, the correlation coefficients between this method and other methods were above 0.504 (*p* < 0.05), and the direct bilirubin (DBIL) of low concentration and the indirect bilirubin (IBIL) of high concentration could not interfere with the detected results of nine indicators. The novel multiplex detection method, which can increase accuracy and improve the ability of comprehensive analysis, can basically meet the requirement of detection and the diagnosis of the proteomics of nutrition and health.

## 1. Introduction

The detections of protein markers of nutrition and health are necessary when evaluating the statuses of nutrition and health [1,2], which mainly involve the statuses of protein, vitamin, mineral element, hyperlipidemia, tumor and so on. With the developing immunologically detective technique, the detection of the protein marker has been applied to more and more research on nutrition and health.

The statuses of the protein, Vitamin A, and iron, as well as cardiovascular, cerebrovascular disease and carcinoma, are five main fields of nutrition and health in China [3], which mainly involve nine protein markers, including pre-albumin (PA), retinol-binding protein 4 (RBP4), serum ferritin (SF), soluble transferrin receptor (sTfR), C-reactive protein (CRP), apolipoprotein B (Apo B), D-Dimer (D-D), alpha-fetoprotein (AFP) and carcinoma embryonic antigen (CEA). Therefore, the detections for the above nine protein markers are the technical basis for researching nutrition and health, especially for nutrition-and-health proteomics.

At present, immunological methods are mainstream ways of detecting the above nine protein markers, which mainly include electro-chemiluminescence immunoassay (ECLIA), radioimmunoassay (RIA), immune turbidimetry, and the enzyme-linked immunosorbent assay (ELISA). They have once appeared with a favorable performance in the previous study at a certain degree, while all of them could not realize multiplex and simultaneous detection for the above nine protein markers. So, the detection efficiency and system error of these conventional methods could not meet the requirements of nutrition-and-health proteomics on a more accurate and panoramic analysis for the above five main fields.

The protein chip technique is an important detection means that is often used to support the data analysis of proteomics, and it is one kind of multiplex and quantitative detection technique which could realize the simultaneous detection of multi-protein markers using the same one detection. According to the different detection conditions, the protein chip includes a solid chip and a liquid chip. The basic detection principle of the protein chip is the known different protein molecules (such as an enzyme, antigen, antibody, ligand and cell factor) are, respectively, which are fixed on the surface of the carrier (solid surface or microsphere) of the protein chip after the carrier is processed as a special chemical method. Then, these protein molecules capture the corresponding target proteins (protein markers) in serum, plasma, lymph, urine and secretion according to the immunological characteristics of these known protein molecules. After that, the quantitative results of these protein markers are used to conduct proteomics analysis. Until now, the protein chip technique has been widely used in many fields of clinical detection, such as the screening of gene expression, the detection of a specific antigen or antibody, the study of the interaction between different proteins, and the research for new medicine [4]. More and more research has verified that the protein chip technique can contribute to research in the proteomics of the clinical field.

Our team constructed multiplex detection methods for protein markers by using solid protein chips in two aspects of the nutritional field. These two explorations aimed to identify the simultaneous detection of SF and sTfR in the serum [5] and the simultaneous detection of β-lactoglobulin and lactoferrin in bovine milk [6]. These results verified that the solid protein chip could be used in multiplex detection for protein markers of nutrition at a certain level but also showed the limitation of this at the technique level, which mainly involved the insufficiently mixed homogeneous phase of a solid chip, which could directly impede the realization of multiplex detection when the indicators were larger than four indicators. This limitation could further lead to the poor accuracy and precision of simultaneous detection. The reason for causing an insufficiently mixed homogeneous phase is that the fixed capture protein molecule on the surface of the solid chip could not fully react with the corresponding protein marker in a static system.

The liquid protein chip is a new-generation biochip and a new type of research platform for protein markers, which can provide technical support for multi-analyte profiling (MAP) immunoassay. In the late 1990s, scientists invented MAP technology, which was a major advance in multiplexed biological assays. This technology drew from the strengths of solid-phase separation technology without the typical limitations of solid-phase reaction kinetics. By combining advanced fluidics, optics, solid chip, and digital signal processing with proprietary microsphere technology, MAP technology enabled a high degree of multiplexing within a single sample volume [7], which could be applied in both proteomics [8] and genomics [9]. As an important branch, the liquid protein chip can realize capture sandwich immunoassay [10], competitive immunoassay [11], indirect immunoassay [12,13], and the combination of capture sandwich and competitive immunoassay. The capture sandwich immunoassay, the main immunoassay of the liquid protein chip, uses a covalent bond that was produced by a carboxyl ammonia reaction to joining with the capture protein molecule on the surface of microbeads (microspheres). The protein molecules that join with microbeads can sufficiently contact the corresponding target protein in serum or other liquid to constitute a dynamically mixed homogeneous phase through the use of a horizontal oscillator. Therefore, the liquid protein chip can overcome the limitation of the insufficiently mixed homogeneous phase of a solid protein chip and, thus, can possess higher sensitivity and accuracy and realize the accurate and simultaneous multiplex detection of multiple protein markers [14]. The multiplex detection of liquid protein technique, which has a relatively low systemic error, low cost, and flexible assembly among different protein markers, can promote the wide application of that which, in the proteomic analysis of many fields, includes the exploration of tumor markers [15], the early diagnosis of Alzheimer’s disease [16], the analysis of virus [17], and other similar research [18,19,20].

Whether the liquid protein chip technique can be used in the detections of the above nine protein markers of nutrition and health to provide a more accurate and comprehensive analysis for proteomics of nutrition and health remains to be seen. The current references do not indicate that any research has attempted to construct a similar method that can simultaneously detect the above nine proteins until now.

Depending on the above analysis and hypothesis, our team adopted the timely liquid protein chip technique to construct a new multiplex detection method for nine protein markers of nutrition and health so as to overcome the limitation of the solid protein chip and to further increase the detective throughput of protein markers in nutrition and health proteomics.

According to our research, we attempted to introduce the construction and evaluation of a novel MAP capture sandwich immunoassay for nine nutrition-and-health-related protein markers based on the liquid protein chip technique in this article, which could provide better technique supports and more comprehensive data for the analysis of the proteomics of nutrition and health in the future. This attempt is a meaningful exploration of the liquid protein chip technique in nutrition and health. Further, we also hope that the nutrition-and-health proteomics based on this constructed method can play more roles in the monitoring and intervention of nutrition and health of large-scale crowds in the future.

## 2. Materials and Methods

### 2.1. Materials and Reagents

All the reagents of this research can be searched from Table 1.

### 2.2. Main Instruments and Equipment

MILLIPLEX Multiplexing Assays (Merck & Co., Inc., Darmstadt, Germany); Millipore-Q Academic (Merck & Co., Inc., Darmstadt, Germany); Allegra x-22R Centrifuge (Beckman coulter, Inc., Brea, CA, USA); SpectraMax I3X Enzyme marker (Molecular Devices Instruments Ltd., San Jose, CA, USA); Desk centrifuge 5418 (Eppendorf, Inc., Hamburg, Germany); Vortex mixing device ORTEx Genius (IKA, Inc., Staufen, Germany); Digital ultrasonic cleaner (Kunshan Ultrasonic Instruments Co., Ltd., Kunshan, China); Bio-Plex Handheld Magnetic Washer (Bio-Rad Laboratories, Inc., Berkeley, CA, USA); Oscillator (Thermo Scientific, Co., Ltd., Waltham, MA, USA); Magnetic Shelf DvnaMag-2 (Thermo Scientific, Co., Ltd., Waltham, MA, USA); Biochemical incubator SPX-150B (Shanghai Pudong Rongfeng Scientific Instrument Ltd., Shanghai, China); 7600 Series Automatic Analyzer (HITACH Diagnostic Product Shanghai Ltd., Shanghai, China); Roche E601 Immunoluminescence instrument (Roche, Inc., Basel, Switzerland).

### 2.3. Serum Samples

The serum samples were obtained from the UNICEF project “Observation on the YYB intervention on health, cognition and behavior”. All guardians agreed with the content of informed consent from the National Institute for Nutrition and Health (NINH) and Chinese Center for Disease Prevention and Control, and the number of NINH ethic committee was (NO. 2018-20). All serum samples were frozen at −80 °C before they were detected.

### 2.4. The Basic Operation Procedure of Liquid Protein Chip

The basic operation procedures of the coupling of the microsphere and the capture of antibodies include the activation of the microsphere with a ProteOn Amine Coupling kit, carbodiimide coupling for antibodies with a Bio-Plex Amine Coupling kit, blocking with a Bio-Plex Amine Coupling kit, and storage with a Bio-Plex Amine Coupling kit. All of these operations were implemented according to the introductions of the ProteOn Amine Coupling kit and Bio-Plex Amine Coupling kit. Additionally, the coupled microspheres with different kinds of capture antibodies were stored at 4 °C.

The basic operation procedures where the antigen combines with a capture antibody include the addition of the coupled microspheres in each well of 96 well bottom plates (3000 microspheres in each well), the rinse of microspheres (100 μL wash buffer, 3 times), the addition of antigens (standard substance or serum) to each well, and horizontal oscillation with 700 rpm (1 h) after 1100 rpm (30 s) (Room temperature and avoid light).

The basic operation procedures where the antigen combines with the detection antibody with biotin include the rinsing of microspheres (100 μL wash buffer, 3 times), the addition of a detection antibody with biotin into each well, and a horizontal oscillation with 700 rpm (30 min) after 1100 rpm (30 s) (at room temperature and avoid light). The basic operation procedures for the detection of the antibody combined with SAPE include the rinsing of microspheres (100 μL wash buffer, 3 times), the addition of 50 μL SAPE in each well, and horizontal oscillation with 700 rpm (15 m) after 1100 rpm (30 s) (at room temperature and avoid light). Additionally, then, microspheres were re-suspended under the condition of 700 rpm at 15–20 min after 1100 rpm (30 s) (at room temperature and avoid light).

**Figure 1:**
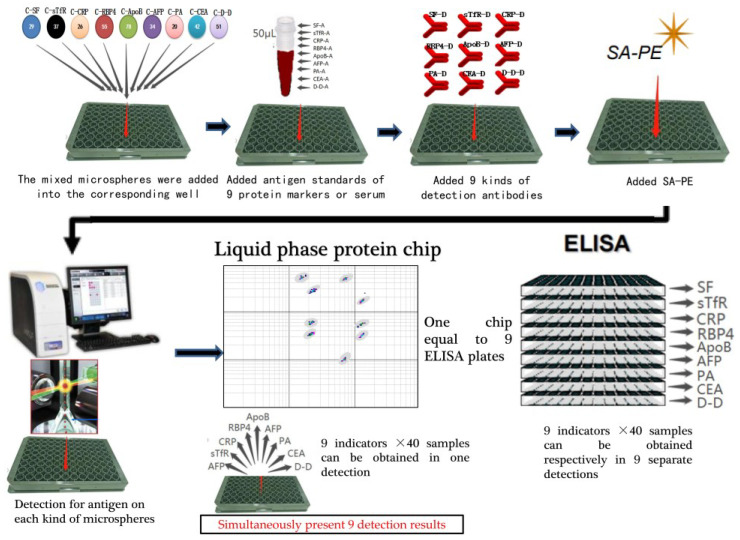
The basic operation procedure of liquid protein chip.

Finally, 9 kinds of antigens that, respectively, combined with the corresponding microsphere can simultaneously be detected, and their signal values could be simultaneously obtained under 532 nm and 635 nm through MILLIPLEX Multiplexing Assays. Figure 1 displays the basic operation procedure.

### 2.5. Optimization of the Combinations of Capture Antibody Concentrations and Detection Antibody Titers of Each Protein Marker

The chessboard titration analysis was adopted to optimize the appropriate concentration of the capture antibody and the titer of the detection antibody of each protein marker. In this method, the optimal concentration of the capture antibody for each protein marker was determined from 3 alternative concentrations (SF, sTfR, CRP, RBP4, AFP and PA: 13.3 μg/mL, 26.6 μg/mL, 53.3 μg/mL, ApoB, CEA and D-D: 26.6 μg/mL, 53.3 μg/mL, 80 μg/mL) according to the pre-experiment of each capture antibody, and the optimal titer of the detection antibody of each protein marker was determined from 3 alternative titers (SF and PA:1:50, 1:100, 1:200, sTfR, AFP, CEA and D-D:1:100, 1:200, 1:400, CRP and RBP4: 1:250, 1:500, 1:1000, ApoB: 1:200, 1:400, 1:800) according to the suggestion of the manufacturer for each detection antibody, and the concentrations of each protein marker included high and low concentrations (SF: 26.00 ng/mL, 2.60 ng/mL, sTfR: 333.33 ng/mL, 10.00 ng/mL, CRP: 800.00 ng/mL, 0.05 ng/mL, RBP4: 14.50 μg/mL, 1.00 μg/mL, ApoB: 125.00 μg/mL, 250.00 ng/mL, AFP: 15.20 μg/mL, 1.52 ng/mL, PA: 50.00 μg/mL, 2.00 ng/mL, CEA: 940.00 pg/mL, 235.00 pg/mL, D-D: 80.00 ng/mL, 4.00 ng/mL) according to the normal range and pre-experiment of each protein marker, and 1% BSA was used as a blank control for each protein marker. The specific operation refers to Section 2.4.

The optimal combination of the capture antibody concentration and detection antibody titer was determined when both the highest signal value and lowest background value simultaneously met under this combination, and the distance value between the high and low concentration of the protein marker was the biggest at this time.

### 2.6. The Verification of Specific Binding Ability between Antigen and Antibody

The cross-reaction experiment was adopted to verify the specific binding ability between the antigen and antibody [5,6]. The concentration of the capture antibody and the titer of the detection antibody for each protein marker depended on the optimal results of Section 2.5. [The capture antibodies of SF, sTfR, RBP4, and AFP were adopted at 53.3 μg/mL, those of CRP and PA adopted at 26.6 μg/mL, and those of ApoB, CEA and D-D were adopted at 80 μg/mL. At the same time, the detection antibodies SF and AFP were adopted 1:100, those of PA, CEA and D-D was adopted 1:200 and those of sTfR and ApoB were adopted 1:400, and those of CRP and RBP4 were adopted 1:1000, respectively. In addition, the concentrations of nine protein markers were SF (26.00 ng/mL), sTfR (333.33 ng/mL), CRP (0.40 μg/mL), RBP4 (10.00 μg/mL), ApoB (25.00 μg/mL), AFP (3.80 μg/mL), PA (50.00 μg/mL), CEA (1.87 ng/mL), and D-D (0.16 μg/mL), respectively]. The blank control used 1% BSA. The specific operation refers to Section 2.4.

There is a specific binding ability between the antigen and antibody if the detection result is significantly bigger than that of the blank control (*p* < 0.05). There is a cross-reaction between one antigen and another antibody (for example, SF and the antibody of sTfR) if the cross-reaction rate of them is larger or equal to 20% of that between this antigen and the antibody itself (for example, SF and the antibody of SF). The cross-reaction rate can be calculated using Formula (1):

*CRR* (%) = (*C*_c_ − *C*_0_) × 100 / (*C*_p_ − *C*_b_) (1)

*CRR*: cross reaction rate,
*C*_c_: reaction value of cross reaction between an antigen and other antibody,
*C*_0_: blank value of cross reaction between an antigen and other antibody,
*C*_p_: reaction value of an antigen and its own antibody,
*C*_b_: blank value of an antigen and its own antibody

### 2.7. Determinations of Lower Limit of Detection and Biologic Limit of Detection

The design of this experiment refers to reference (Sensitivity for Analysis) [21], and the mixed antigen standard of 9 protein markers was diluted 8 times using serial 2 multiple dilutions within the range of a low concentration, which meant that the mixed antigen standard included SF, sTfR, CRP, RBP4, ApoB, AFP, PA, CEA, and D-D was diluted from the high point (1.63 ng/mL, 0.33 ng/mL, 3.13 ng/mL, 7.08 ng/mL, 30.50 ng/mL, 1.48 ng/mL, 12.21 ng/mL, 1.88 ng/mL, 2.50 ng/mL) to a low point (6.30 pg/mL, 1.30 pg/mL, 12.20 pg/mL, 27.70 pg/mL, 119.20 pg/mL, 6.00 pg/mL, 47.70 pg/mL, 7.30 pg/mL, 9.80 pg/mL). The concentration of the capture antibody and the titer of the detection antibody for each protein marker depended on the optimal results of Section 2.5. (The capture antibodies of SF, sTfR, RBP4 and AFP adopted 53.3 μg/mL, and those of CRP and PA adopted 26.6 μg/mL, and those of ApoB, CEA and D-D adopted 80 μg/mL. At the same time, the detection antibodies of SF and AFP adopted 1:100, those of PA, CEA and D-D adopted 1:200, those of sTfR and ApoB adopted 1:400, and those of CRP and RBP4 adopted 1:1000), respectively. The blank control used 1% BSA. The specific operation refers to Section 2.4.

The corresponding concentration value of the twofold standard deviation (2SD) of the signal value of a blank well is the Lower Limit of Detection (LLD), and the concentration value is the Biologic Limit of Detection (BLD) when it is just larger than LLD after it subtracts 2SD.

### 2.8. The Establishments of S-Curves and the Determination of Regression Equation

The mixed standard substance of 9 protein markers was successively diluted 20 times using serial 2 multiple dilutions, which meant that the mixed standard substance that included SF, sTfR, CRP, RBP4, ApoB, AFP, PA, CEA and D-D was successively diluted from a high mixed point (6.66 μg/mL, 1.33 μg/mL, 12.8 μg/mL, 29.00 μg/mL, 125.00 μg/mL, 6.08 μg/mL, 50.00 μg/mL, 7.70 μg/mL and 10.24 μg/mL) to a low mixed point (6.30 pg/mL, 1.30 pg/mL, 12.20 pg/mL, 27.70 pg/mL, 119.20 pg/mL, 6.00 pg/mL, 47.70 pg/mL, 7.30 pg/mL and 9.80 pg/mL). The concentration of the capture antibody and the titer of the detection antibody for each protein marker depended on the optimal results of Section 2.5. The blank well used 1% BSA. The specific operation refers to Section 2.4.

The concentration and the signal value of each protein marker in the mixed system appeared on the *X*-axis and *Y*-axis, respectively, and then, the *S*-curve of each protein marker could be drawn according to the relationship between the concentration and signal value.

After that, the 6 standard points of each protein marker in the mixed system were determined according to the range of the straight linear of the corresponding *S*-curve of each protein marker. Then, the mixed standard substance of SF, sTfR, CRP, RBP4, ApoB, AFP, PA, CEA and D-D was successively diluted from high mixed point (26.00 ng/mL, 333.33 ng/mL, 50.60 ng/mL, 1812.50 ng/mL, 3906.30 ng/mL, 1.48 ng/mL, 12,500.00 ng/mL, 1.88 ng/mL and 320.00 ng/mL) to low mixed point (0.20 ng/mL, 2.60 ng/mL, 0.78 ng/mL, 28.32 ng/mL, 30.52 ng/mL, 0.01 ng/mL, 97.66 ng/mL, 0.01 ng/mL and 2.50 ng/mL) as doubling dilution. At last, 9 standard regression equations for the 9 protein markers of the mixed system were simultaneously established depending on the above 6 standard points of each protein marker.

The determination coefficient of each regression equation should be bigger than 0.95 at least.

### 2.9. Methodological Evaluation and Verification: Accuracy

The adding standard recovery was used to evaluate accuracy according to the “Guidance on the Verification of Quantitative Measurement Procedures used in the Clinical Chemistry (CNAS-GL037, 2019), abbreviation is Guidance”. Two random serums were diluted, respectively at 1:40 (serum I) and 1:100 (serum II), and the mixed antigen standard (SF: 50.05 ng/mL, sTfR: 529.10 ng/mL, CRP: 90.18 ng/mL, RBP4: 1652.42 ng/mL, ApoB: 14,245.01 ng/mL, AFP: 2.16 ng/mL, PA: 21,164.02 ng/mL, CEA: 1.80 ng/mL and D-D: 450.87 ng/mL) was added, respectively, into serum I and serum II as the 4% of basic serum volume (50 μL) and 5% of basic serum volume (50 μL). The standard curve of each protein marker refers to Section 2.8. The blank well used 1% BSA. The specific operation refers to Section 2.4.

The adding standard recovery rate was calculated through Formula (2).

(2) $$R=\frac{C\times (V_{0}+V){-C}_{0}\times V_{0}}{V\times C_{s}}\times 100\%$$

*R*: adding standard recovery, *V*: the volume after standard was added in basic serum, *V*_0_: the volume of basic serum, *C*: the detected concentration after standard was added in basic serum, *C*_0_: the detected concentration of basic serum, *C*_s_: the concentration of standard of each protein marker.

The 100.0% ± 30.0 is an appropriate accuracy range for adding a standard recovery of each protein marker of the mixed system [5].

### 2.10. Methodological Evaluation and Verification: Precision

The precision evaluation included within-run precision and between-run precision. The operations refer to “Guidance”. Two random serums were diluted after at 1:60 and were, respectively, detected with 10 replicates in one day in order to conduct the analysis of within-run precision for this novel method. Another two random serums, which were used to perform a between-run precision, were stored at −20 °C after they were, respectively, divided into 6 aliquot tubes, and freeze–thaw cycles should be avoided. The whole experiment period of between-run precision was 6 days, and 2 serums for each day were, respectively, diluted at 1:60, and they were, respectively, detected with 3 replicates. The standard curve of each protein marker refers to Section 2.8. The blank well used 1% BSA. The specific operation refers to Section 2.4.

The coefficient of variation (CV) in of the detection results of each protein marker was adopted to evaluate the precision of this method. The within-run precision and the between-run precision of this method should be, respectively less than 10% and 20%.

### 2.11. Methodological Evaluation and Verification: Comparison with Reference Method

The operation refers to “Guidance”. A total of 16 random serum samples were, respectively, aliquot into 10 groups, and one group was used to detect 9 protein markers simultaneously through one detection using the liquid protein chip, and four groups were used to detect sTfR, CRP, ApoB and PA, respectively, in four separate experiments through the use of the ELISA method, and 3 groups were used to detect respectively SF, RBP4 and D-D, respectively, in 3 separate experiments using the immunoturbidimetry method, and 2 groups were used to detect AFP and CEA, respectively, in 2 separate experiment using the ECLIA method.

The 16 serum samples, which were used to detect 9 protein markers simultaneously in one detection using the liquid protein chip, were detected after they were diluted at 1:60, and the standard curve of each protein marker refers to Section 2.8. The blank well used 1% BSA. The specific operation is referred to in Section 2.4. The detections of the serums for the other 9 groups, which were detected by using other three detection methods, referred, respectively, to the specification of the detection kit of each protein marker.

For 9 protein markers of the same 16 serum samples, the detected results using a liquid protein chip were compared, respectively, with that of using a separate detection method. The result of the correlation analysis between two different methods should be significant (*p* < 0.05), while the result of the pair comparison between two different methods should not be significant (*p* > 0.05).

### 2.12. Methodological Evaluation and Verification: Analytical Specificity

The operation refers to “Guidance”. Direct bilirubin (DBIL), indirect bilirubin (IBIL), hemoglobin (Hb) and triglycerides (TG) were adopted to judge whether the detection results of the liquid protein chip could be interfered by jaundice, hemolysis and lipoidemia.

Two random serum samples were aliquoted into 9 groups after they were diluted as 1:60. The 2 diluted serum samples of the first group (control group) were used to directly detect the concentrations of 9 protein markers, and those of the second and third group (jaundice I group) were used to detect the concentrations of 9 protein markers after they were added, respectively, into high and low concentration DBIL (28.00 mmol/L, 5.70 μmol/L). Those of the fourth and fifth groups (jaundice II group) were used to detect the concentrations of 9 protein markers after they were added, respectively, into high- and low-concentration IBIL (53.00 mmol/L, 0.48 μmol/L). Those of the sixth and seventh groups (hemolysis group) were used to detect respectively the concentrations of 9 protein markers after they added interfered, respectively, into high and low concentration Hb (160.00 g/L, 71.11 g/L), and those of the eighth and ninth group (lipoidemia group) were used to detect, respectively, the concentrations of 9 protein markers after they were added, respectively, into high and low concentration TG (616.67 μmol/L, 33.33 μmol/L). The dilution of 1% BSA was used, and each case was repeated 3 times. The standard curve of each protein marker refers to Section 2.8. The blank well used 1% BSA. The specific operation refers to Section 2.4.

The mean values for the concentration of the control group and other groups were analyzed, respectively, and the difference value between each experiment group and control group were further calculated. Additionally, the interference rate was calculated by relative bias as Formula (3).

I = D × 100 / C(3)

I: interference rate, D: the difference value of mean values between each experiment group and control group, C: the mean value of control group

The intervention would affect the analytical specificity of liquid protein chip if the interference rate was larger than or equal to 20%.

### 2.13. Statistical Analysis

SPSS19.0 (IBM, Armonk, NY, USA) was used for statistical analysis. In the research, at least three replicated tests were performed in the liquid protein chip. All the data were presented as the means ± standard deviation (SD). The least square method was used to construct the regression equation, and the paired *t*-test or Wilcoxon matching pair symbol rank sum test and the correlation analysis were adopted, respectively, to conduct a comparison with the reference method, and the correlation coefficient was analyzed by a *t*-test (*p* < 0.05). Statistical graphs were produced with Microsoft Excel 2010 (Mircosoft Inc., Redmond, WA, USA).

## 3. Results

### 3.1. Optimization of the Combination of the Capture Antibody Concentration and the Detection Antibody Titer of Each Protein Marker

The result of the optimal combination of the capture antibody concentration and the detection antibody titer for each protein marker indicated that the optimal concentration of the capture antibody and the optimal titer of the detection antibody of SF were 53.3 μg/mL and 1:100, those of sTfR were 53.3 μg/mL and 1:400, those of CRP were 26.6 μg/mL and 1:1000, those of RBP4 were 53.3 μg/mL and 1:1000, those of ApoB were 80.0 μg/mL and 1:400, those of AFP was 53.3 μg/mL and 1:100, those of PA were 26.6 μg/mL and 1:200, those of CEA was 80.0 μg/mL and 1:200, and those of D-D were 80.0 μg/mL and 1:200, respectively.

The detection conditions of the subsequent experiment depended on the parameters of the above optimal combination.

### 3.2. The Verification of Specific Binding Ability between Antigen and Antibody

The cross-reaction rates of each protein marker and different antibodies appear in Table 2 and Table 3. The results indicate that the range of the cross-reaction rates of each protein marker and non-detection antibody was 0.00–4.05%, and that of each protein marker and non-capture antibody was 0.00–9.96%. The results of cross-reaction could meet the requirement that performs the multiplex and simultaneous detection for nine protein markers using the liquid protein chip.

### 3.3. Determinations of Lower Limit of Detection and Biologic Limit of Detection

The LLD and BLD of each protein marker are shown in Table 4, and the LLD range of that liquid protein chip and simultaneously detected nine protein markers was between 0.90 and 214.20 pg/mL, and the BLD range of that was between 2.55 and 1900.00 pg/mL.

### 3.4. The Establishments of S-Curves and the Determination of Regression Equation

**Figure 2:**
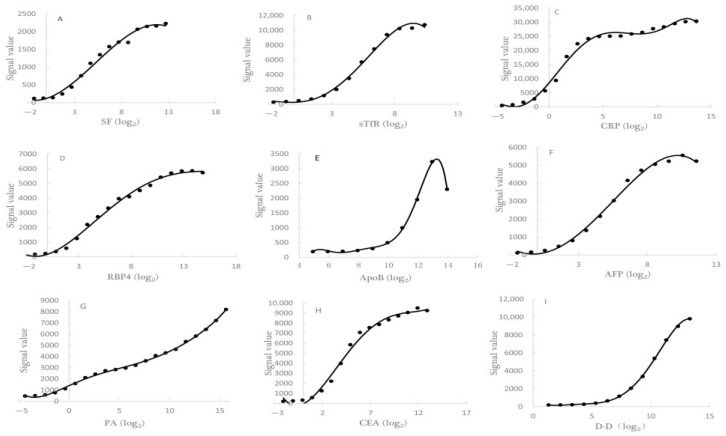
The *S*-curves of 9 protein markers in the same one detection by using liquid protein chip. (**A**): SF *S*-curve; (**B**): sTfR *S*-curve; (**C**): CRP *S*-curve; (**D**): RBP4 *S*-curve; (**E**): ApoB *S*-curve; (**F**): AFP *S*-curve; (**G**): PA *S*-curve; (**H**): CEA *S*-curve; (**I**): D-D *S*-curve.

The S-curves of nine protein markers, which were simultaneously detected by using the liquid protein chip in the same detection, displayed in Figure 2, and the linear ranges of the nine protein markers were, respectively, SF (0.20~26.00 ng/mL), sTfR (2.60~333.30 ng/mL), CRP (0.40~50.00 ng/mL), RBP4 (7.08~1812.50 ng/mL), ApoB (30.50~3906.25 ng/mL), AFP (0.01~1.48 ng/mL), PA (0.10~13.80 μg/mL), CEA (0.01~1.88 ng/mL) and D-D (2.50~320.00 ng/mL).

The regression equations and determination coefficients for the nine protein markers that were simultaneously detected using a liquid protein chip in the same detection are in Table 5.

The detection method of the liquid protein chip could simultaneously detect nine protein markers in the same detection, which had been constructed until now.

### 3.5. Methodological Evaluation and Verification: Accuracy

The accuracy range of the nine protein markers was between 70.12 and 127.07%, and the accuracy of each protein marker is shown in Table 6.

### 3.6. Methodological Evaluation and Verification: Precision

The precision ranges of the within-run precision and between-run precision were, respectively, 0.85–7.31% and 3.53–19.07%. The specific results are shown in Table 7 and Table 8, respectively.

### 3.7. Methodological Evaluation and Verification: Comparison with Reference Method

**Figure 3:**
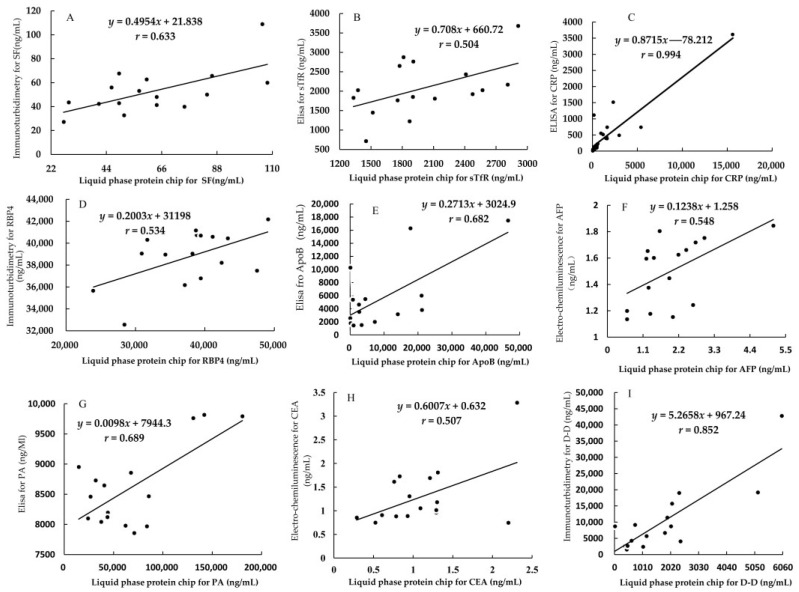
Correlation analysis between liquid protein chip method and other methods for detecting the 9 protein markers of the same 16 serum samples. (**A**): The correlation analysis of SF; (**B**): The correlation analysis of sTfR; (**C**): The correlation analysis of CRP; (**D**): The correlation analysis of RBP4; (**E**): The correlation analysis of ApoB; (**F**): The correlation analysis of AFP; (**G**): The correlation analysis of PA; (**H**): The correlation analysis of CEA; (**I**): The correlation analysis of D-D.

The results indicate that the correlation coefficient of each protein marker between the liquid protein chip and another corresponding detection method was significant (*p* < 0.05) and are shown in Figure 3 and Table 9.

In addition, the results of the pair comparisons indicated that there was no significant difference between any of the liquid protein chips and other corresponding detection methods (*p* > 0.05), which are shown in Table 10.

### 3.8. Methodological Evaluation and Verification: Analytical Specificity

The results indicate that a low concentration of DBIL (28.00 nmol/L) and a high and low concentration of IBIL (0.48 μmol/L, 53.00 nmol/L) would not interfere with the detection results of nine protein markers. In addition, the high concentration of DBIL (5.70 μmol/L), the high and low concentration of TG (616.67 μmol/L, 33.33 μmol/L), and the high and low concentration of Hb (160.00 g/L, 71.11 g/L) could interfere with the detection results of the liquid protein chip to a certain of degree. Table 11 displays the specific results of analytical specificity.

## 4. Discussion

The liquid protein chip is a core technique of proteomics, which can provide technical support for the systematic research of a field when different protein indicators are needed to explain a phenomenon [22]. Our research is an exploration of this technique in nutrition and health.

As we know, SF, sTfR and CRP [23,24,25] can be used to judge the iron level, PA [26] can be used to judge the protein level, RBP4 [27] can be used to judge the VA level, ApoB [28] and D-D [29] can be used to reflect the risk of cardiovascular and cerebrovascular disease, AFP [30] is a marker of liver cancer development and progression, and CEA [31] is a preferred indicator of colon-cancer staging studies before surgical resection. The system error of the conventionally separate detection for each indicator above might disturb the correct analysis for the relationship among the nine protein markers. Therefore, there is a growing need to construct a new multiplex detection method that can realize a comprehensive judgement of the relationship among them. Our team has long engaged in the research of analyzing the relationship between serum indicators and the health of the human body, and the purpose of this research is was construct and evaluate a new method that could perform multiplex detection for the above nine protein markers at the same detection so as to provide support for comprehensive observation and analysis at the viewpoint of nutrition-and-health proteomics at the next stage of our research.

The cross-reaction rate could directly affect the accuracy and recovery rate of the protein marker. Although it is very important for accuracy, there is not a uniform and mature threshold value standard in the field of liquid protein detection. Therefore, we had no choice but to set 10% and 20% as the relative threshold values of the cross-reaction rate according to the requirement of “Guidance on the Verification” on accuracy and the results of our team on other similar research [5]. Considering this requirement, we considered the cross-reaction to be nonexistent when the cross-reaction rate was less than 10%, to be weak when the rate was between 10% and 20%, and not tolerated when the rate was larger than 20%. In this research, the results of the cross-reaction rates for the nine protein markers indicated that all of them were less than 10%, which meant there was no cross-reaction in the constructed method.

The LLD results indicate that the LLDs of liquid protein chip on eight protein markers (SF, sTfR, CRP, RBP4, ApoB, AFP, CEA and D-D) were, respectively, lower than those of the comparison methods, which appeared in the preponderance of the liquid protein chip for the detection of these indicators. Our team had constructed a method for the solid protein chip to detect SF and sTfR simultaneously, and the comparative results indicated that the LLDs of the liquid protein chip were obviously better than those of the solid protein chip when simultaneously detecting SF and sTfR, which further proved that the performance of liquid protein chip was better than that of solid protein chip. The comparisons among the above different methods can be found in Table 12.

Referred to as “Guidance on the Verification of Quantitative Measurement Procedures used in the Clinical Chemistry (CNAS-GL037, 2019)”, we preliminarily completed the evaluation of the new detection method from four aspects, including accuracy, precision, a comparison with the reference method, and analytic specificity. For the accuracy of this method, the evaluated result indicated that it met the basic requirements of our research at the preliminary stage, especially at the early application stage of the new technique, because this method took into consideration all factors of the nine protein markers and realized the synergy of detecting nine protein markers in the same detection; additionally, the collection of comprehensive and systematic information in one detection is more important for the research of nutrition-and-health proteomics. In the evaluation of accuracy, the recovery rates of CRP, AFP, PA and CEA exceeded 100%, which reasons that the operation errors of adding standard substances are larger or the specificities of the antibodies are relatively weak. Therefore, we need to further control the operation of experimenters and choose better antibodies with better specificity in the next stage of research so as to improve the recovery rate.

In comparison with the reference method, the reason that we did not choose the same method as the reference methods for detecting different protein markers is there is no golden standard in the field of detecting protein, so we had to adopt the common method of aiming at each protein marker as a reference method for each protein marker. Therefore, ELISA was used as a comparison method for the liquid protein chip to detect sTfR, CRP, ApoB and PA, Immunoturbidimetry was used as a comparison method for the detection of SF, RBP4 and D-D, and ECLIA was used as comparison methods to detect AFP and CEA according to the practical conditions of our routine detections. In addition, although eight samples were enough according to the requirement of the comparison within the reference method of “Guidance on the Verification of Quantitative Measurement Procedures used in the Clinical Chemistry (CNAS-GL037, 2019)”, we still randomly selected 16 samples to perform this evaluation. The reason for this is that we wanted to verify the feasibility of our method as fully as possible. The results indicate that our method was competent in the comparison of each conventional method. Although the diagnostic efficiency of our method is equal to single detection, our method is able to produce more comprehensive information that could contribute to analyzing the relationship among these indicators from the viewpoint of proteomics. In addition, we also can provide support to nutrition-and-health proteomics by using this method so as to explore the relationship between nutrition and health in the next stage of our research.

In the evaluation of analytical specificity, the results indicate that the sample should not be icterus, lipoidemia and hemolysis at a certain degree, which means that the detection result of our method might be disturbed by some disease factors. In the next stage, we need to further improve our method by optimizing the detection condition so as to improve the applicability of this method.

At present, more and more researchers are paying more and more attention to the detection of nutrition- and health-related protein markers. Our detection method realized the simultaneous detection of nine protein markers of each of the 40 samples within 5 h, which could not only decline a system error [32] but could also shorten the detection time, reduce cost and increase detection efficiency. Table 13 shows the specific comparisons of the detection performance between the liquid protein chip method and other methods, which indicate the superiority of our method in practice detection. On the other hand, the comparison with other similar methods on the basis of liquid protein ships indicates that our method has basically the same detection time, close cost and similar detection efficiency because the basic principles of them are absolutely the same [33,34,35].

For the comparison of multiplex and single plex protein detection, the core of the controversy is actually the diagnostic value, which is appropriate between combined diagnosis on the basis of combined detection and the combined diagnosis on the basis of single detection for the same sample. Referring to the discussion of accuracy above, our research has proved that the diagnostic value of the combined diagnosis on the basis of combined detection (multiplex detection) is equal to that of the basis of single detection, while the results of multiplex detection could provide more comprehensive and accurate information for nutrition-and-health proteomics in one detection.

On the other hand, the detective throughput is one key point of the comparison between different multiplex detection methods, which directly influences the overall performance of a multiplex detection method. The throughput of different research is generally different, which depends on the purpose of each research. At the present, although the theoretic throughput of the MAP immunoassay technology can reach 50 protein markers [7], the range of the throughput is commonly from 2-plex to 30-plex in practical applications [33,34,35]. Therefore, compared with other multiplex methods [33,36,37,38,39], the throughput of our method is moderate, which means that the analysis time, the cost, and the technical requirements of our methods are basically in moderation with practical applications. Thus, the comparison also means that there is great potential in the multiplex detection of nutrition-and-health proteomics, which we need to devote more effort to in order to enhance the throughput by attempting more protein markers.

We hope our novel MAP immunoassay method can gradually be used in the comprehensive assessment of nutrition and health and the monitoring of the large-scale population so as to provide technical support for real-time and effective prevention or for the accurate intervention of nutrition and health.

## 5. Conclusions

Based on the liquid protein chip technique, a novel multiplex detection method for nine nutrition-and-health-related protein markers was successfully constructed after we determined the optimal combination of the concentrations of the capture antibody and detection antibody for each protein marker, determined the cross-reaction rate of each protein marker, and determined the LLD and BLD of each protein marker through a series of optimal experiments. At the same time, the evaluated results of the accuracy, precision, and comparison with the reference method and analytical specificity have indicated that this novel method can basically meet the requirement of the laboratory and can preliminarily replace conventional methods. Based on the above analysis and discussion, the constructed novel method based on a liquid protein chip is a worthy exploration in the fields of nutrition and health, which might provide more support for nutrition-and-health proteomics, and might be used gradually in the comprehensive assessment of nutrition and the health of large-scale populations in the future.

## Figures and Tables

**Table 1:** Reagents used in this research.

The Kind of Reagent	The Name of Product	The Code of Product	Company (City, State, Country)
Basic reagents	Phosphate buffer (PBS)	KG018279	Jiangsu KeyGEN BioTECH Co., Ltd. (Nanjing, China)
	SAPE	S866	Thermo fisher Co., Ltd. (Waltham, MA, USA)
	Bovine serum albumin (BSA)	WXBC7961V	Sigma Co., Ltd. (St. Louis, MO, USA)
Coupling reagent	ProteOn Amine Coupling kit	1762410	Bio-Rad Laboratories, Inc. (Berkeley, CA, USA)
	Bio-Plex Amine Coupling kit	171-406001	Bio-Rad Laboratories, Inc. (Berkeley, CA, USA)
Antigen standards, capture antibodies and detection antibodies	SF antigen standard	30-AF15	Fitzgerald Industries International, Inc. (Acton, MA, USA)
	SF capture antibody	MAB4062	R&D Inc. (Minneapolis, MN, USA)
	SF detection antibody	NB110-8384B	Novus Biologicals, Inc. (Centennial, CO, USA)
	sTfR antigen standard	05-52172	ARP, Inc. (Waltham, MA, USA)
	sTfR capture antibody	ab10249	Abcam, Inc. (Cambridge, UK)
	sTfR detection antiboty	NB100-73092B	Novus Biologicals, Inc. (Centennial, CO, USA)
	C-reaction protein standard	30-AC05AF	Fitzgerald Industries International, Inc. (Acton, MA, USA)
	C-reaction capture antibody	MAB17071	R&D Inc. (Minneapolis, MN, USA)
	C-reaction detection antibody	BAM17072	R&D Inc. (Minneapolis, MN, USA)
	RBP4 antigen standard	30-1358	Fitzgerald Industries International, Inc. (Acton, MA, USA)
	RBP4 capture antibody	MAB33781	R&D Inc. (Minneapolis, MN, USA)
	RBP4 detection antibody	BAM33782	R&D Inc. (Minneapolis, MN, USA)
	Apo B antigen standard	SRP6302-500UG	Sigma Co., Ltd. (St. Louis, MO, USA)
	Apo B capture antibody	A2299-38D	USbiological Inc. (Salem, MA, USA)
	Apo B detection antibody	A2299-38E	USbiological Inc. (Salem, MA, USA)
	PA antigen standard	30R-AP014	Fitzgerald Industries International, Inc. (Acton, MA, USA)
	PA capture antibody	MAB7505	R&D Inc. (Minneapolis, MN, USA)
	PA detection antibody	NBP2-90050B	Novus Biologicals, Inc. (Centennial, CO, USA)
	AFP antigen standard	PRO-406	ProSpec-Tany TechnoGene Ltd. (Ness-Ziona, Israel)
	AFP capture antibody	NB110-7961	Novus Biologicals, Inc. (Centennial, CO, USA)
	AFP detection antibody	NB120-10072B	Novus Biologicals, Inc. (Centennial, CO, USA)
	CEA antigen standard	30C-CP1001	Fitzgerald Industries International, Inc. (Acton, MA, USA)
	CEA capture antibody	10-C10F	Fitzgerald Industries International, Inc. (Acton, MA, USA)
	CEA detection antibody	orb14352	Biorbyt, Inc. (Cambridge, UK)
	D-D antigen standard	D9321	Sigma Co., Ltd. (St. Louis, MO, USA)
	D-D capture antibody	NB110-8376	Novus Biologicals, Inc. (Centennial, CO, USA)
	D-D detection antibody	NB100-73038B	Novus Biologicals, Inc. (Centennial, CO, USA)
Interference reagents	Direct bilirubin	14370-250MG	Sigma Co., Ltd. (St. Louis, MO, USA)
	Indirect bilirubin	F1618-B	FANKE WEI, Inc. (Shanghai, China)
	Hemoglobin	H7379	Sigma Co., Ltd. (St. Louis, MO, USA)
	Triglyceride	G107438-5g	Aladdin, Inc. (Shanghai, China)
Comparison Kits	SF immunoturbidimetry kit	FE7002	Beijing Leadman, Inc. (Beijing, China)
	sTfR ELISA kit	SEKH-0330	Beijing Solarbio life science, Inc. (Beijing, China)
	C-reaction protein ELISA kit	SEKH-0138	Beijing Solarbio life science, Inc. (Beijing, China)
	Apo B ELISA kit	SEKH-0515	Beijing Solarbio life science, Inc. (Beijing, China)
	PA ELISA kit	EK1684	Wuhan Boster Biological Technology, Ltd. (Wuhan, China)
	RBP4 immunoturbidimetry kit	RB7001	Beijing Leadman, Inc. (Beijing, China)
	D-D immunoturbidimetry kit	DD7680	Beijing Leadman, Inc. (Beijing, China)
	AFP ECLIA kit	4481798190	Roche diagnostic product, Inc. (Shanghai, China)
	CEA ECLIA kit	11731629322	Roche diagnostic product, Inc. (Shanghai, China)

**Table 2:** The cross-reaction rates of 9 protein markers with each detection antibody (%).

Antigen	Detection Antibody								
	SF-D	sTfR-D	CRP-D	RBP4-D	ApoB-D	AFP-D	PA-D	CEA-D	D-D-D
SF	100.00	−0.18	0.01	1.22	−0.11	−0.04	−0.25	−7.27	0.00
sTfR	−4.87	100.00	0.00	1.61	−0.14	−0.04	0.04	0.17	−0.10
CRP	−0.59	0.09	100.00	−0.66	0.03	1.09	0.00	−0.99	0.00
RBP4	−1.16	0.18	0.07	100.00	0.06	0.00	0.02	−0.25	0.00
ApoB	−2.59	−0.22	−0.03	0.28	100.00	0.31	0.08	4.05	0.20
AFP	0.19	−0.02	0.01	−0.07	0.00	100.00	−0.02	−0.33	0.00
PA	0.74	0.00	−0.04	0.13	−0.09	−0.03	100.00	0.74	0.03
CEA	−1.99	0.02	0.00	−0.02	0.00	0.05	−0.08	100.00	−0.39
D-D	1.09	0.02	0.00	0.00	0.00	0.67	0.00	−0.08	100.00

**Table 3:** The cross-reaction rates of 9 protein markers with each capture antibody (%).

Antigen	Capture Antibody								
	C-SF	C-sTfR	C-CRP	C-RBP4	C-ApoB	C-AFP	C-PA	C-CEA	C-D-D
SF	100.00	−14.39	−0.48	−5.35	0.18	−0.15	0.24	−1.40	−1.41
sTfR	0.00	100.00	0.15	0.52	0.37	0.06	0.15	1.74	2.02
CRP	0.00	−0.13	100.00	0.21	0.00	0.00	0.02	0.00	0.00
RBP4	0.04	0.02	0.09	100.00	0.21	0.06	0.04	0.08	0.10
ApoB	4.99	9.96	1.02	4.78	100.00	3.32	7.36	2.07	2.52
AFP	0.08	0.09	0.40	0.49	−0.31	100.00	0.89	0.33	0.20
PA	0.04	0.00	0.06	5.17	0.11	0.04	100.00	0.08	0.20
CEA	0.17	0.00	0.00	0.00	−0.08	−0.02	0.00	100.00	4.30
D-D	0.00	0.00	0.01	0.02	0.47	0.03	0.09	0.00	100.00

**Table 4:** The LLDs and BDLs of liquid protein chip for 9 protein markers (pg/mL).

Limit of Detection	SF	sTfR	CRP	RBP4	ApoB	AFP	PA	CEA	D-D
LLD	12.70	1.00	0.90	45.20	134.10	5.40	21.70	6.20	214.20
BLD	25.40	2.55	12.20	55.30	1900.00	12.50	47.70	7.30	312.50

**Table 5:** The regression equations and determination coefficients of 9 protein markers detected by the liquid protein chip.

Protein Marker	Regression Equation	Determination Coefficients (*r*^2^)
SF	*y* = −0.9106*x*^2^ + 63.867*x* − 40.79	0.997
sTfR	*y* = 0.0012*x*^3^ − 0.7202*x*^2^ + 139.45*x* + 433.6	0.992
CRP	*y =* −0.2861*x*^4^ + 26.523*x*^3^ − 758.02*x*^2^ + 7860*x* − 573.33	0.998
RBP4	*y* = 619.95ln(*x*) + 621.12	0.996
ApoB	*y* = 0.467*x* − 80.259	0.995
AFP	*y* = 119.54*x* + 0.6148	0.997
PA	*y* = 660.51 ln(*x*) − 395.57	0.981
CEA	*y* = 30.35*x*^2^ + 164.82*x* + 9.9296	0.998
D-D	*y* = 5.9577*x* − 6.648	0.999

**Table 6:** The recovery rates of 9 protein markers detected by liquid protein chip simultaneously (*n* = 3).

Serum Cases	SF	sTfR	CRP	RBP4	ApoB	AFP	PA	CEA	D-D
Serum I + 4% mixed antigen standard	79.66	94.7	105.98	82.66	91.51	109.46	87.25	103.37	77.53
Serum II + 5% mixed antigen standard	70.12	74.91	121.3	85.5	72.63	122.77	127.07	85.72	86.12

**Table 7:** The within-run precisions of 9 protein markers by liquid protein chip (*n* = 10).

Serum Cases	Evaluation Indicators	Protein Markers								
		SF	sTfR	CRP	RBP4	ApoB	AFP	PA	CEA	D-D
Serum I	$\overset{-}{x}$ (ng/mL)	0.25	64.87	10.18	152.60	29.09	0.50	134.12	0.25	51.02
	*S* (ng/mL)	0.01	2.31	0.72	4.68	1.79	0.03	1.15	0.02	1.14
	*CV* (%)	4.42	3.57	7.11	3.07	6.15	4.87	0.85	7.31	2.23
Serum II	$\overset{-}{x}$ (ng/mL)	0.36	52.12	8.81	155.94	61.87	2.37	130.83	1.48	226.69
	*S* (ng/mL)	0.02	1.22	0.30	8.38	4.13	0.17	2.18	0.08	6.90
	*CV* (%)	6.38	2.35	3.35	5.38	6.68	6.95	1.67	5.37	3.04

**Table 8:** The between-run precisions of 9 protein markers by liquid protein chip (*D* = 6, *n* = 3).

Serum Cases	Evaluation Indicators	Protein Markers								
		SF	sTfR	CRP	RBP4	ApoB	AFP	PA	CEA	D-D
Serum I	$\overset{-}{x}$ (ng/mL)	0.23	21.70	1.55	13.98	50.79	0.02	144.65	0.01	7.77
	*Sb* ^2^	0.00	4.43	0.04	1.65	74.50	0.00	69.38	0.00	0.52
	*CV* (%)	6.66	9.70	12.08	9.19	16.99	10.12	5.76	4.85	9.28
Serum II	$\overset{-}{x}$ (ng/mL)	0.23	20.76	3.31	13.08	31.62	0.02	122.44	0.01	18.69
	*Sb* ^2^	0.00	15.67	0.27	1.20	6.44	0.00	18.65	0.00	4.96
	*CV* (%)	16.55	19.07	15.74	8.39	8.03	8.45	3.53	3.65	11.92

**Table 9:** The correlation analysis between the liquid protein chip method and other methods for the detection of 9 protein markers in the same 16 serums (*n* = 16).

Protein Markers	Between Liquid Protein Chip and Immunoturbidimetry		Between Liquid Protein Chip and ECLIA		Between Liquid Protein Chip and Elisa	
	*r*	*p*	*r*	*p*	*r*	*p*
SF	0.633	0.008	—	—	—	—
sTfR	—	—	—	—	0.504	0.047
CRP	—	—	—	—	0.994	<0.001
RBP4	0.534	0.033	—	—	—	—
ApoB	—	—	—	—	0.682	0.004
AFP	—	—	0.548	0.028	—	—
PA	—	—	—	—	0.689	0.003
CEA	—	—	0.507	0.045	—	—
D-D	0.852	<0.001	—	—	—	—

Note: “—” means this method did not compare with liquid protein chip at this indicator.

**Table 10:** The paired comparison between liquid protein chip method and other methods for the detection of 9 protein markers of serums (*n* = 16).

Statistic Indicators	Between Liquid Protein Chip and ECLIA		Between Liquid Protein Chip and Immunoturbidimetry			Between Liquid Protein Chip and Elisa			
	AFP	CEA	SF	RBP4	D-D	sTfR	CRP	ApoB	PA
*t*	—	0.127	0.951	−0.675	—	−0.943	—	—	—
*p*	0.079	0.901	0.356	0.510	0.877	0.360	0.070	0.756	0.501

Note: “—” means this comparison did not meet the requirement of paired *t*-test, which adopted Wilcoxon matching pair symbol rank sum test.

**Table 11:** The analytical specificities of different interferences for the liquid protein chip in detecting 9 protein markers (%).

Interferent	CRP	SF	AFP	sTfR	D-D	RBP4	PA	CEA	ApoB
DBIL (high)	−17.27	34.08	−10.74	−18.49	86.22	−10.90	1.05	−3.66	−10.91
DBIL (low)	10.76	6.58	0.20	−0.38	4.76	−7.21	−2.78	2.88	−9.32
IBIL (high)	10.07	13.00	4.34	0.58	−6.47	−15.33	−4.87	8.86	−18.69
IBIL (low)	16.41	4.19	15.21	2.82	8.56	−17.17	−6.67	13.15	−18.41
TG (high)	29.27	21.95	25.71	21.08	39.73	−0.86	1.12	38.02	−27.69
TG (low)	49.84	11.84	17.25	5.79	28.81	−1.94	−2.59	11.80	−22.90
Hb (high)	−5.96	3.90	−21.38	−4.06	−20.75	−3.23	2.55	−8.92	−32.09
Hb (low)	37.07	23.10	31.53	22.23	42.87	3.48	−0.53	62.06	−30.67

**Table 12:** The comparison of the LLDs of 9 protein markers between liquid protein chip and other methods.

Protein Markers	Liquid Protein Chip (pg/mL)	Solid Protein Chip (ng/mL)	ELISA (pg/mL)	Immunoturbidimetry (μg/mL)	ECLIA (ng/mL)
SF	12.70	1.21 ^a^	—	0.40 ^e^	
sTfR	1.00	1.73 ^a^	18.00 ^b^	—	—
CRP	0.90	—	5.36 ^b^	—	—
RBP4	45.20	—	—	50.00 ^d^	—
ApoB	134.10	—	3910.00 ^b^	—	—
AFP	5.40	—	—	—	0.50 ^f^
PA	21.70	—	6.30 ^c^	—	—
CEA	6.20	—	—	—	0.50 ^f^
D-D	214.20	—	—	0.50 ^d^	—

Note: ^a^, data from reference [5]; ^b^, data from ELISA kit specification of Beijing Solarbio life science, Inc. (Beijing, China); ^c^, data from ELISA kit specification of Wuhan Boster Biological Technology., LTD (Wuhan, China); ^d^ and ^e^, data from immunoturbidimetry kit specification of Beijing Leadman, Inc. (Beijing, China); ^f^, data from electro-chemiluminescence kit specification of Roche diagnostic product, Inc. (Shanghai, China). —, our research did not adopt this method to perform Section 2.11.

**Table 13:** Comparison between our method and other methods.

Detection Methods	Throughput	Volume of Detecting 9 Indicators (μL)	The time of Detecting 9 Indicators (h)	Cost of Single Indicator of Each Sample (CNY)	Whether the Results Can Be Appeared in the Same One Detection	The System Error of Detecting 9 Indicators
Liquid protein chip	9	2	5	6.75	Yes	Smaller
ELISA ^a^	1	36	45	100	No	Larger
Immunoturbidimetry ^b^	1	150	3	50	No	Larger
ECLIA ^c^	1	150	3	50	No	Larger

Note: ^a^, was the common method in detecting sTfR, CRP, ApoB and PA; ^b^, was the common method in detecting SF, RBP4 and D-D; ^c^, was the common method in detecting AFP and CEA. The data of above other three methods referred to the information of kit specification of each method.

## Data Availability

The data supporting the reported results can be obtained from the corresponding author.

## References

[1] Rosalind S.G. (2005). Principles of Nutritional Assessment.

[2] Jorg R., Albert S. (2009). Proteomics: Methods and Protocols.

[3] (2020). Report on Chinese Residents’ Chronic Diseases and Nutrition (2020) (English Version).

[4] Duarte J.G., Blackburn J.M. (2017). Advances in the development of human protein microarrays. Expert Rev. Proteom..

[5] Yin J.Y., Sun J., Huang J., Li W., Huo J.S. (2012). Study on the method of quantitative analysis of serum ferritin and soluble transferrin receptor with protein microarray technology. Biomed. Environ. Sci..

[6] Yin J.Y., Huo J.S., Ma X.X., Sun J., Huang J. (2017). Study on the Simultaneously Quantitative Detection for β-Lactoglobulin and Lactoferrin of Cow Milk by Using Protein Chip Technique. Biomed. Environ. Sci..

[7] Stephen A., Shubhagata D., Wilco G., Sherry D. xMAP Cookbook. https://f.hubspotusercontent30.net/hubfs/128032/Cookbook/BR402139.xMAPCookbook.Ed5.All.Sections.WR.pdf.

[8] Syu G.D., Dunn J., Zhu H. (2020). Development and application of functional protein microarrays. Mol. Cell. Proteom..

[9] Wu C., Chen M., Zhang Q., Yu L., Zhu J., Gao X. (2019). Genomic and genechip expression profiling reveals the inhibitory effects of amorphophalli rhizome in TNBC cell. J. Ethnopharmacol..

[10] Francois P.M., David J., Sebastien B. (2021). Precise chip-to chip reagent transfer for cross reactivity-free multiplex sandwich immunoassays. Methods Mol. Biol..

[11] Goldman E. (2008). TNT detection using llama antibodies and a two-step competitive fluid array immunoassay. J. Immunol. Methods.

[12] Yuan L., Jianhua H., Jing L., Wentao F., Zhenwei B., Suquan S., Liping Y. (2021). Development and application of a novel triplex protein microarray method for rapid detection of antibodies against avian influenza virus, Newcastle disease virus, and avian infectious bronchitis virus. Arch. Virol..

[13] Huanan W., Feng C., Jianchi G., Li X., Yujun Z., Yuexiao L., Ren H., Meili C., Pengju G. (2019). Establishment of xMAP for the simultaneous detection of antibodies to Newcastle disease virus and avian influenza virus. Poult. Sci..

[14] Jitka M., Karel C., Jiri P., Jiri K., Pavla R., Ondrej S., Pavel T. (2018). Simultaneous detection of chicken cytokines in plasma samples using the Bio-Plex assay. Poult. Sci..

[15] Sun G., Ye H., Wang X., Cheng L., Ren P., Shi J., Dai L., Wang P., Zhang J. (2020). Identification of novel autoantibodies based on the protein chip encoded by cancer-driving genes in detection of esophageal squamous cell carcinoma. Oncoimmunology..

[16] Erika O.H., Natalia S.D., Ivonne C.B.B., Monica V.C., Andrea T.C., Antonio L.T., Izabela G.B., Lorena A.V.S., Daniela V.F.R., Aloisio J.F.R. (2021). Millipore xMap^®^ Luminex (HATMAG-68K): An Accurate and Cost-Effective Method for Evaluating Alzheimer’s Biomarkers in Cerebrospinal Fluid. Front. Psychiatry.

[17] Cristina A., Tamara R., Linda D., Sandra B., Paloma R., Patricia S. (2019). Bead-Based Multiplex Assay for the Simultaneous Detection of Antibodies to African Swine Fever Virus and Classical Swine Fever Virus. Front. Vet. Sci..

[18] Lei Z., Xiao W., Cheng Z., Antonio F.L., Mei-hhua Y. (2018). A Review of Current Methods for Analysis of Mycotoxins in Herbal Medicines. Toxins.

[19] Hall S.A., Ison S.H., Owles C., Coe J., Sandercock D.A., Zanella A.J. (2019). Development and validation of a multiplex fluorescent microsphere immunoassay assay for detection of porcine cytokines. MethodsX.

[20] Houman M., Walid M.H., Yasmine D., Emma G., Jan J.D., Azam F.T. (2022). Rapid, Sensitive, and Selective Quantification of Bacillus cereus Spores Using xMAP Technology. Microorganism.

[21] Davenport J.M., Schlain B. (2000). Testing claimed minimal detectable concentrations of in vitro medical diagnostic devices. Clin. Chem..

[22] Wang H., Li H., Zhang W., Wei L., Yu H., Yang P. (2014). Multiplex profiling of glycoproteins using a novel bead-based lectin array. Proteomics.

[23] Ahmed H.S. (2022). Prevalence of anemia among children in India and updated serum ferritin levels. Pediatr. Blood Cancer.

[24] Suvi K., Cecile S., Victor N., Leisel T., Andre B., Christian R., Henrik F., Pernille K. (2020). Vitamin A and iron status of children before and after treatment of uncomplicated severe acute malnutrition. Clin. Nutr..

[25] Julia L.F., Anura V.K., Beena B., Tinku T., Krishnamachari S., Christopher D. (2020). Anaemia and iron deficiency in pregnancy and adverse perinatal outcomes in Southern India. Eur. J. Clin. Nutr..

[26] Ruvini N.R., Milly B., Royce P.V. (2022). Prealbumin: The clinical utility and analytical methodologies. Ann. Clin. Biochem..

[27] Vasily M.S., Baptiste W., Marco N., Christel C., Aline A., Camille A., Celine D., Serge S., Jose-Alain S., Christina Z. (2022). Large benefit from simple things: High doe vitamin A improves RBP4-related retinal dystrophy. Int. J. Mol. Sci..

[28] Melinda E.T., Brigitta D., Zsofia H., Miklos S. (2020). Cerebrovascular changes and neurodegeneration related to hyperlipidemia: Characteristics of the human ApoB-100 transgenic mice. Curr. Pharm. Des..

[29] Ohara T., Farhoudi M., Bang O.Y., Koga M., Demchuk A.M. (2020). The emerging value of serum D-dimer measurement in the work-up and management of ischemic stroke. Int. J. Stroke.

[30] Javier S., Anne L. (2021). The heterogeneity of liver cancer metabolism. Adv. Exp. Med. Biol..

[31] Saran L., Alejandro R.B., Burt C. (2022). Colon Cancer. Treasure Island.

[32] Christopher P., Thomas E. (2020). Multiplex Immunoassay Techniques for On-Site Detection of Security Sensitive Toxins. Toxins.

[33] Merck Biomarker Detection Solution Scheme. https://www.sigmaaldrich.cn/deepweb/assets/sigmaaldrich/marketing/global/documents/417/008/biology-mark-test-solution.pdf.

[34] Stine R.R., Sakers A.P., Teslaa T., Kissig M., Seale P. (2019). PRDM16 Maintains Homeostasis of the Intestinal Epithelium by Controlling Region-Specific Metabolism. Cell Stem Cell.

[35] Häussler R.S., Bendes A., Iglesias M.J., Sanchez-rivera L., Dodig-crnkovic T., Bystrom S., Fredolini C., Birqersson E., Dale M., Edfors F. (2019). Systematic development of sandwich immunoassays for the plasma secretome. Proteomics.

[36] Schenk M.F., Cordewener J.H.G., America A.H.P., Peters J., Smulders M.J.M., Gilissen L.J.W.J. (2011). Proteomic analysis of the major birch allergen Bet v 1 predicts allergenicity for 15 birch species. J. Proteom..

[37] Campbell C.T., Llewellyn S.R., Thorsten D., Morgan I.L., Marjorie R.G., Gildersleeve J.C., Shan L. (2013). High-throughput profiling of anti-glycan humoral responses to SIV vaccination and challenge. PLoS ONE.

[38] Kirouac D.C., Du J.Y., Lahdenranta J., Overland R., Yarar D., Paragas V., Pace E., Mcdonagh C.F., Nielsen U.B., Onsum M.D. (2013). Computational modeling of ERBB2-amplified breast cancer identifies combined ErbB2/3 blockade as superior to the combination of MEK and AKT inhibitors. Sci. Signal..

[39] Meimaridou A., Haasnoot W., Shelver W.L., Franek M., Nielen M.W.F. (2013). Multiplex immunoassay for persistent organic pollutants in tilapia: Comparison of imaging- and flow cytometry-based platforms using spectrally encoded paramagnetic microspheres. Food Addit. Contam. Part A.

